# Association of Stress Hyperglycemia Ratio With Acute Ischemic Stroke Outcomes Post-thrombolysis

**DOI:** 10.3389/fneur.2021.785428

**Published:** 2022-01-13

**Authors:** Chuan-Li Shen, Nian-Ge Xia, Hong Wang, Wan-Li Zhang

**Affiliations:** ^1^Department of Ultrasonography, The First Affiliated Hospital of Wenzhou Medical University, Wenzhou, China; ^2^Department of Neurology, The First Affiliated Hospital of Wenzhou Medical University, Wenzhou, China

**Keywords:** ischemic stroke, stress hyperglycemia, thrombolysis, outcomes, stress hyperglycemia ratio

## Abstract

**Background and Purpose:** The association between stress hyperglycemia and clinical outcomes in patients with acute ischemic stroke treated with intravenous thrombolysis (IVT) is uncertain. We sought to analyze the association between the stress hyperglycemia ratio (SHR) using different definitions and clinical outcomes in acute patients with ischemic stroke undergoing IVT.

**Methods:** A total of 341 patients with ischemic stroke receiving IVT were prospectively enrolled in this study. The SHR was evaluated using different equations: SHR1, fasting glucose (mmol/L)/glycated hemoglobin (HbA1c) (%); SHR2, fasting glucose (mmol/L)/[(1.59 × HbA1c)−2.59]; SHR3, admission blood glucose (mmol/L)/[(1.59 × HbA1c)−2.59]. A poor functional outcome was defined as a modified Rankin scale score of 3–6 at 3 months. Multivariate logistic regression analysis was used to identify the relationship between different SHRs and clinical outcomes after IVT.

**Results:** A total of 127 (37.2%) patients presented with poor functional outcomes at 3 months. The predictive value of SHR1 for poor functional outcomes was better than that of SHR2 and SHR3 in receiver operating characteristic analyses. On multivariate analysis, SHR1 [odds ratio (OR) 14.639, 95% CI, 4.075–52.589; *P* = 0.000] and SHR2 (OR, 19.700; 95% CI; 4.475–86.722; *P* = 0.000) were independently associated with an increased risk of poor functional outcome but not SHR3.

**Conclusions:** Our study confirmed that the SHR, as measured by SHR1 and SHR2, is independently associated with worse clinical outcomes in patients with ischemic stroke after intravenous thrombolysis. Furthermore, SHR1 has a better predictive performance for outcomes than other SHR definitions.

## Introduction

Acute ischemic stroke is associated with high mortality and disability rates (1, 2). Intravenous thrombolysis with alteplase within 4.5 h of the onset of acute ischemic stroke is the safest and most effective treatment recommended by the guidelines (3, 4). Several studies have investigated the prognostic factors of intravenous thrombolytic therapy for ischemic stroke (5–8), among which the serum glucose level has attracted the attention of many researchers (9).

Several studies have demonstrated that hyperglycemia is independently associated with poor outcomes and symptomatic intracerebral hemorrhage (SICH) in patients with thrombolyzed AIS (7, 10, 11). However, a potential disparity exists in the association between hyperglycemia and poor outcomes when stratified by the history of diabetes mellitus (12, 13). Hyperglycemia was independently associated with mortality and SICH following intravenous thrombolysis in patients without diabetes but not in patients with diabetes (13). Known diabetes can affect the association between hyperglycemia and clinical outcomes. None of the clinical trials that target serum glucose levels have been proved to improve the prognosis of ischemic stroke (14, 15). We may need to find new indicators to replace serum glucose as a treatment target to obtain better clinical trial results.

Recently, the stress hyperglycemia ratio, which is defined as the ratio of blood glucose to glycated hemoglobin (HbA1c), has been considered a novel marker of worse outcomes in patients with ischemic stroke after intravenous thrombolysis (16, 17). However, the definitions of SHR using other equations also predicted poor outcomes in patients with ischemic stroke or critical illness (18, 19). Moreover, the potential mechanism of these phenomena is not fully understood. No studies have explored the effect of different kinds of SHR on the prognosis of patients with acute ischemic stroke. Therefore, the objective of the present study was to determine whether SHR using different definitions was associated with the clinical outcomes of patients with acute ischemic stroke undergoing intravenous thrombolysis.

## Materials and Methods

### Study Population

We performed a prospective, observational study from March 2013 to May 2019 in which we collected data from consecutive patients with AIS undergoing intravenous thrombolysis at the First Affiliated Hospital of Wenzhou Medical University. Each patient was assessed by an experienced neurologist, and the eligible ones received intravenous alteplase within 4.5 h of the stroke onset. The inclusion criteria for enrollment were as follows: 1. Age ≥18 years, 2. The diagnosis of acute ischemic stroke met the World Health Organization's criteria (20), 3. An onset-to-treatment time within 4.5 h after the stroke onset. The exclusion criteria were in accordance with the 2013 AHA/ASA Guidelines (21). In addition, 73 normal healthy subjects were included in the control group. The study protocol was approved by the ethics committee of the First Affiliated Hospital of Wenzhou Medical University (No. KY2021-R077). All the patients or their representatives provided written informed consent.

### Clinical Protocol and Laboratory Tests

On admission and at the time point of 24 h after treatment, the severity of neurological deficits using the National Institutes of Health Stroke Scale (NIHSS) scores was assessed by certified neurologists. We also collected baseline data on age, sex, clinical laboratory findings, onset-to-treatment time, admission blood pressure, medical history, including potential risk factors in stroke, and 12-lead electrocardiography during hospitalization. The blood glucose level on admission had been rapidly measured before the treatment. A medical history of diabetes mellitus was determined based on previous physicians' diagnoses and the use of antidiabetic drugs. All the participants routinely underwent computed tomographic scans at admission and repeated imaging 24 h after thrombolysis, or any other time when the patient experienced neurological deterioration.

Fasting glucose and 2-h postprandial blood glucose were collected during the morning hours (range: 06:00 AM−10:00 AM) after overnight fasting within 24 h after intravenous thrombolysis. HbA1c levels were routinely measured within 48 h after hospitalization. SHR was evaluated using the following equations: SHR1, fasting glucose (mmol/L)/HbA1c (%) (16); SHR2, fasting glucose (mmol/L)/[(1.59 × HbA1c)−2.59] (18); SHR3, admission blood glucose (mmol/L)/[(1.59 × HbA1c)−2.59] (19). The attending physicians and follow-up staff were blinded to the SHR, which was calculated only after all the information had been acquired.

### Outcome Measures

Our primary endpoint of all patients was a poor functional outcome, which was defined as a modified Rankin Scale (mRS) score of 3–6 at the 3-month follow-up appointment. The mRS score was obtained *via* the outpatient department or by a telephone follow-up interview. The secondary outcomes included early neurological improvement (ENI), death within 3 months of follow-up, and intracerebral hemorrhage. ENI was defined as an NIHSS score improvement of ≥ 8 from the baseline or decreased to 0 or 1 at 24 h after treatment (22). The definition of **symptomatic intracerebral hemorrhage (SICH)** was based on the Safe Implementation of Thrombolysis in Stroke-Monitoring Study protocol (23): Type 2 parenchymal hemorrhage that occurred within 24 h after treatment, combined with an increase of ≥ 4 points in the NIHSS score.

### Statistical Analyses

All analyses were conducted using SPSS Statistics (version 24.0; SPSS Inc., Chicago, IL, USA). The threshold for statistical significance was set at *p* < 0.05. Continuous variables were expressed as means with standard deviations or medians with interquartile ranges. Categorical variables are presented as frequencies and percentages. Statistical analyses were performed using Student's *t*-tests, one-way ANOVA test, and the Kruskal-Wallis test for continuous variables, and the chi-square test or Fisher's exact test for categorical variables. Receiver operating characteristic (ROC) curves were constructed to determine the predictive value of admission blood glucose, fasting plasma glucose, HbA1c, SHR1, SHR2, and SHR3 for a poor functional outcome at 3 months. The established optimal cutoff was used to transform SHR1 into a categorical variable. According to the optimal cutoff, we divided all the patients into two groups and compared their baseline characteristics. We employed different multivariate logistic regression models to identify the associations between SHR1, SHR2, SHR3, and outcome measures at 3 months.

## Results

### Baseline Characteristics

**Figure 1:**
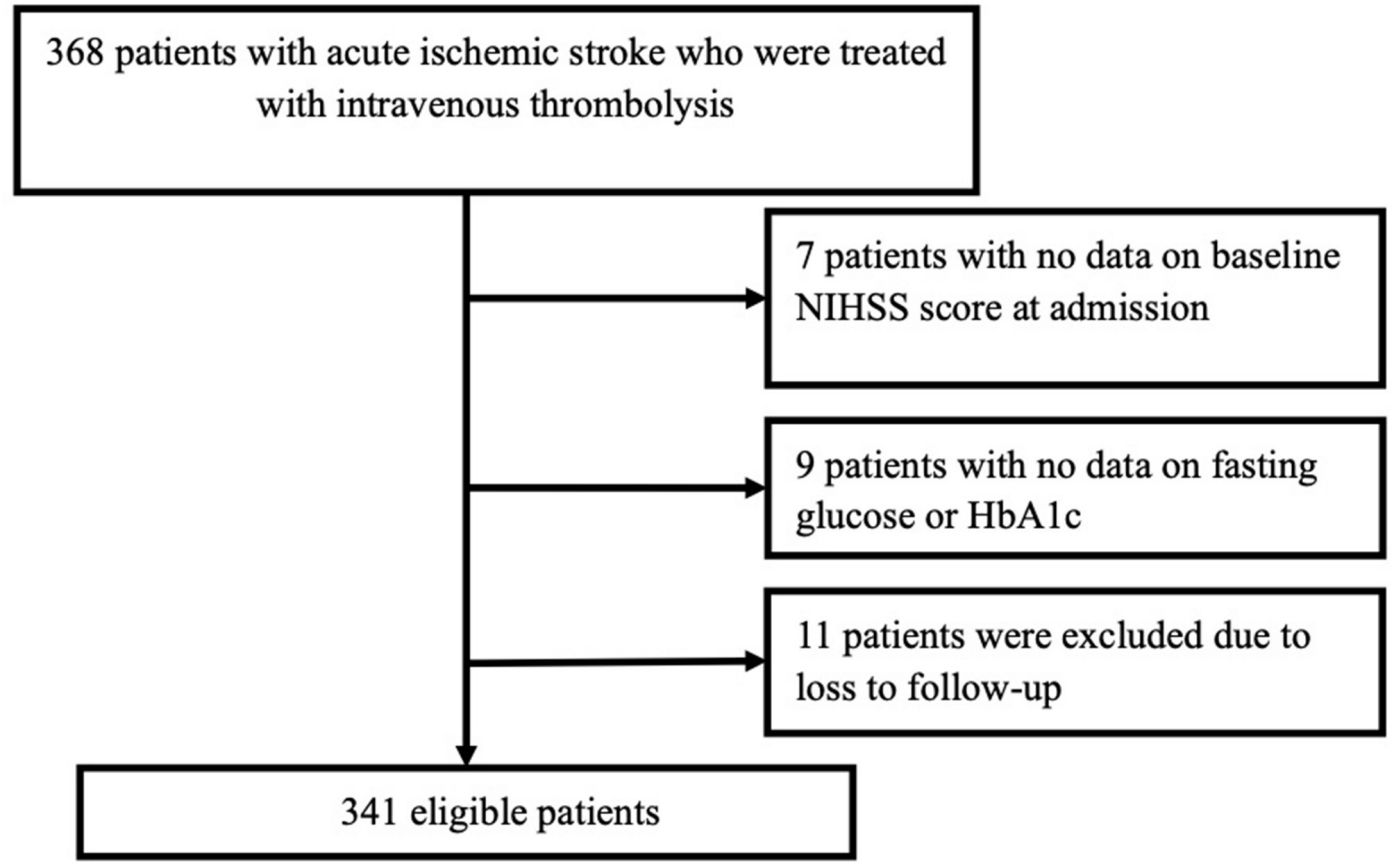
A flow diagram showing the patient selection process.

After excluding patients with missing data for fasting glucose or HbA1c, those with no data on the NIHSS score at admission, and those lost to follow-up at 3 months, we identified 341 eligible patients in the present study (Figure 1). The general clinical characteristics of the subjects are presented in Table 1. The mean age of the included patients was 66.4 ± 12.6 years, and 70.7% of them were males. Among the 341 patients, 26 (7.6%) died during the 3 months, 10 (2.9%) presented with SICH, 69 (20.2%) experienced ENI, and 127 (37.2%) experienced poor functional outcomes. The baseline and clinical characteristics of the patients stratified according to the primary outcomes are summarized in Table 1. The patients with poor functional outcomes had higher NIHSS scores on admission, SHR1, and SHR2 than the patients with good functional outcomes.

**Table 1:** Comparison of the baseline characteristics between patients with poor and good functional outcomes.

**Characteristics**	**Total** **(***n*** = 341)**	**Good outcome** **(***n*** = 214)**	**Poor outcome** **(***n*** = 127)**	***P***
Age (y), mean ± SD	66.4 ± 12.6	63.3 ± 11.8	71.6 ± 12.1	0.000
Gender, male, *n* (%)	241 (70.7)	157 (73.4)	84 (66.1)	0.157
Baseline NIHSS score, median (IQR)	7 (4, 11)	6 (3, 9)	11 (7, 15)	0.000
24 h NIHSS score, median (IQR)	5 (2, 11)	3 (2, 6)	11 (7,16)	0.000
History of smoking, *n* (%)	139 (40.8)	98 (45.8)	41 (32.3)	0.014
Coronary artery disease, *n* (%)	36 (10.6)	21 (9.8)	15 (11.8)	0.562
Hypertension, *n* (%)	229 (67.2)	134 (62.6)	95 (74.8)	0.021
Diabetes, *n* (%)	77 (22.6)	49 (22.9)	28 (22.0)	0.856
Hyperlipidemia, *n* (%)	119 (34.9)	69 (32.2)	50 (39.4)	0.182
Previous stroke/TIA, *n* (%)	41 (12.0)	23 (10.7)	18 (14.2)	0.347
Atrial fibrillation, *n* (%)	85 (24.9)	38 (17.8)	47 (37.0)	0.000
Systolic BP (mmHg), mean ± SD	155.3 ± 23.3	153.7 ± 22.4	158.1 ± 24.5	0.088
Diastolic BP (mmHg), mean ± SD	86.1 ± 15.2	86.2 ± 14.9	85.9 ± 15.7	0.855
Admission blood glucose (mmol/L), mean ± SD	8.0 ± 3.2	7.7 ± 3.1	8.3 ± 3.2	0.098
Fasting plasma glucose (mmol/L), mean ± SD	6.2 ± 2.5	5.8 ± 2.1	6.9 ± 3.0	0.000
2-h postprandial blood glucose	8.3 ± 3.4	8.3 ± 3.5	8.5 ± 3.2	0.666
HbA1c (%), mean ± SD	6.4 ± 1.5	6.3 ± 1.4	6.5 ± 1.6	0.169
SHR1, mean ± SD	0.96 ± 0.24	0.91 ± 0.19	1.05 ± 0.29	0.000
SHR2, mean ± SD	0.83 ± 0.20	0.78 ± 0.16	0.90 ± 0.23	0.000
SHR3, mean ± SD	1.07 ± 0.26	1.05 ± 0.25	1.09 ± 0.28	0.154
Onset to treatment time, min, mean ± SD	203.8 ± 63.0	202.4 ± 63.0	206.3 ± 63.1	0.583
Cell count at admission				
WBC, x10∧9/L, mean ± SD	8.2 ± 2.8	8.1 ± 2.8	8.1 ± 2.7	0.784
RBC, x10∧12/L, mean ± SD	4.6 ± 0.6	4.6 ± 0.5	4.5 ± 0.6	0.016
PLT, x10∧9/L, mean ± SD	210.3 ± 67.2	214.4 ± 68.1	203.4 ± 65.4	0.152

*NIHSS, National Institute of Health Stroke Scale; TIA, transient ischemic attack; BP, blood pressure; HbA1c, glycated hemoglobin; SHR, **s**tress hyperglycemia ratio; WBC, white blood cell; RBC, red blood cell; PLT, platelet; SD, standard deviation; IQR, interquartile range*.

Supplementary Table S1 also shows the comparison of baseline characteristics between the normal, good outcome, and poor outcome groups. The good outcome group and the poor outcome group showed higher levels of SHR1 and SHR2 than the normal group.

### ROC Curve Evaluating the Predictive Value of SHR for Outcomes

Receiver operating characteristic curve analysis was used to evaluate the predictive values of admission blood glucose, fasting plasma glucose, HbA1c, SHR1, SHR2, and SHR3 for 3-month poor functional outcomes (Table 2). The predictive value of SHR1 for clinical outcomes was better than that of admission blood glucose, HbA1c, fasting plasma glucose, SHR2, and SHR3. The area under the curve (AUC) of SHR1 was 0.671 (95% CI,0.611–0.731; *P* = 0.000), and the optimized cut-off of SHR1 value was 0.98.

**Table 2:** Receiver operating characteristic curves, identifying the predictive value of admission blood glucose, fasting plasma glucose, HbA1c, and SHR for poor outcomes at 3 months.

	**AUC (95% CI)**	***p*** **-value**
Admission blood glucose	0.574 (0.511–0.637)	0.023
Fasting plasma glucose	0.654 (0.593–0.714)	0.000
HbA1c	0.544 (0.481–0.606)	0.178
SHR1	0.671 (0.611–0.731)	0.000
SHR2	0.663 (0.602–0.724)	0.000
SHR3	0.549 (0.485–0.612)	0.138

*HbA1c, glycated hemoglobin; SHR**, s**tress hyperglycemia ratio; AUC, area under the curve; CI, confidence interval*.

### Association Between SHR and Clinical Outcomes

**Figure 2:**
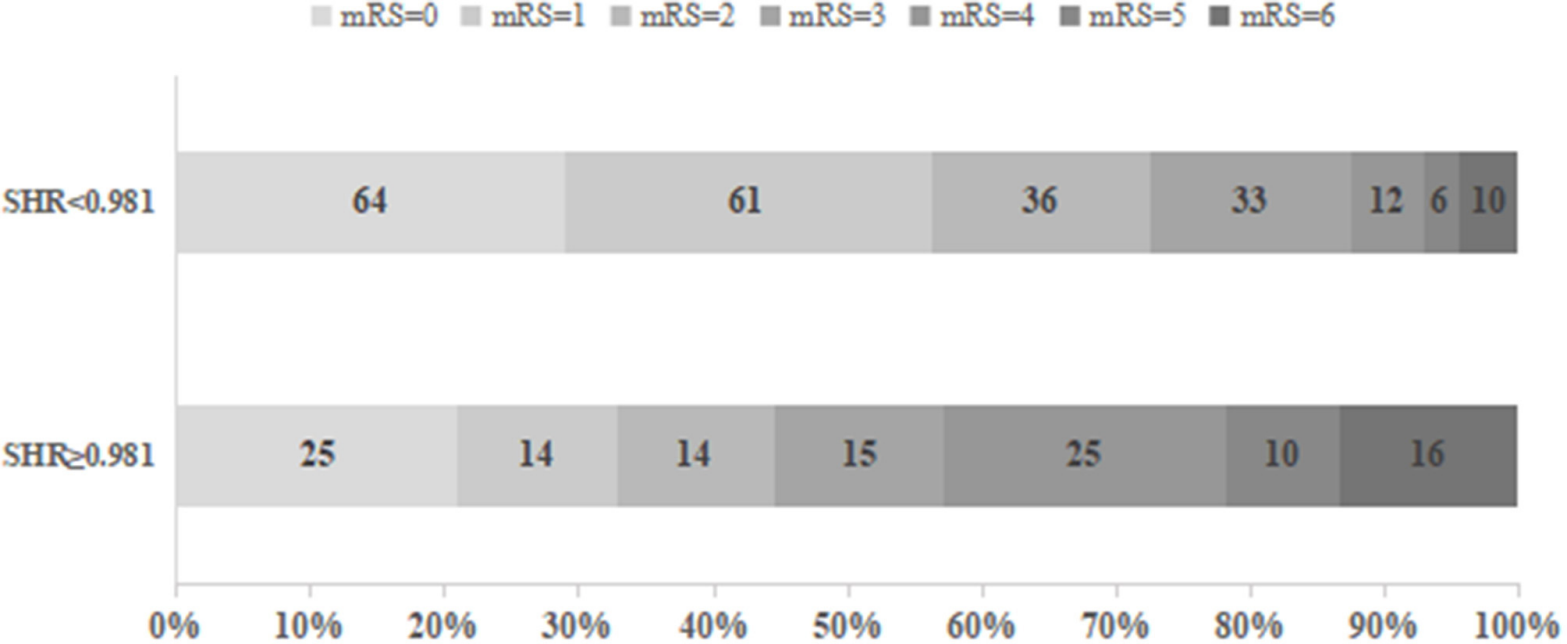
Functional outcomes at 3 months in groups stratified according to the cutoff value of SHR1.

As presented in Table 3, the patients with an SHR1 of ≥0.98 had higher NIHSS scores on admission and at 24 h after intravenous thrombolysis. This group also had a smaller number of smokers and higher prevalence of diabetes mellitus and hypertension compared to the group of patients with an SHR1 of < 0.98. Systolic blood pressure, diastolic blood pressure, and admission blood glucose were significantly higher in patients in the SHR1 ≥0.98 group than in the SHR1 < 0.98 group. The distribution of the mRS score at 3 months stratified by the SHR1 cutoff value is presented in Figure 2. A poor functional outcome and hemorrhagic transformation were more frequent in the higher SHR1 group but less likely to have ENI (Table 3). Table 4 shows the results of the binary logistic regression analysis of the different SHRs and outcome measures. Regardless of whether Model 1 or Model 2 was used, we observed that SHR1 and SHR2 were independently associated with all the outcomes but not SHR3.

**Table 3:** Comparison of the baseline characteristics and outcomes between subgroups stratified according to the SHR1 cutoff.

**Characteristics**	**SHR1≥0.98** **(***n*** =119)**	**SHR1 <0.98** **(***n*** =222)**	***P***
Age (y), mean ± SD	67.3 ± 12.6	65.9 ± 12.6	0.323
Gender, male, *n* (%)	78 (65.5)	163 (73.4)	0.128
Baseline NIHSS score, median (IQR)	10 (5, 14)	7 (4, 10)	0.000
24 h NIHSS score, median (IQR)	9 (4, 13)	4 (2, 9)	0.000
History of smoking, *n* (%)	35 (29.4)	104 (46.8)	0.002
Coronary artery disease, *n* (%)	11 (9.2)	25 (11.3)	0.563
Hypertension, *n* (%)	88 (73.9)	141 (63.5)	0.050
Diabetes, *n* (%)	39 (32.8)	38 (17.1)	0.001
Hyperlipidemia, *n* (%)	44 (37.0)	75 (33.8)	0.556
Previous stroke/TIA, *n* (%)	18 (15.1)	23 (10.4)	0.197
Atrial fibrillation, *n* (%)	36 (30.3)	49 (22.1)	0.096
Systolic BP (mmHg), mean ± SD	158.9 ± 24.5	153.4 ± 22.5	0.041
Diastolic BP (mmHg), mean ± SD	89.8 ± 15.9	84.0 ± 14.4	0.001
Admission blood glucose (mmol/L), mean ± SD	9.0 ± 3.4	7.4 ± 2.9	0.000
Fasting plasma glucose (mmol/L), mean ± SD	8.2 ± 3.1	5.1 ± 1.3	0.000
HbA1c (%), mean ± SD	6.7 ± 1.7	6.2 ± 1.3	0.001
Onset to treatment time, min, mean ± SD	205.4 ± 64.2	203.0 ± 62.5	0.733
Cell count at admission			
WBC, x10∧9/L, mean ± SD	8.3 ± 2.7	8.1 ± 2.8	0.424
RBC, x10∧12/L, mean ± SD	4.5 ± 0.6	4.6 ± 0.5	0.510
PLT, x10∧9/L, mean ± SD	215.8 ± 78.1	207.4 ± 60.7	0.274
Outcomes			
ENI, *n* (%)	13 (10.9)	56 (25.2)	0.002
mRS score, median (IQR)	3 (1, 4)	1 (0, 3)	0.000
mRS score of 3–6, *n* (%)	66 (55.5)	61 (27.5)	0.000
Hemorrhagic transformation, *n* (%)	33 (27.7)	22 (9.9)	0.000
SICH, *n* (%)	6 (5.0)	4 (1.8)	0.176
Death, *n* (%)	16 (13.4)	10 (4.5)	0.003

*SHR**, s**tress hyperglycemia ratio; NIHSS, National Institute of Health Stroke Scale; TIA, transient ischemic attack; BP, blood pressure; HbA1c, glycated hemoglobin; WBC, white blood cell; RBC, red blood cell; PLT, platelet; ENI, early neurological improvement; mRS, modified Rankin Scale; SICH, symptomatic intracerebral hemorrhage; SD, standard deviation; IQR, interquartile range*.

**Table 4:** Multivariate logistic regression analysis of the associations between SHR and outcomes.

	**Multivariate adjusted model** **(Model 1)**		***P***	**Multivariate adjusted model (Model 2)**		***P***
	**OR**	**95% CI**		**OR**	**95% CI**	
Poor outcome						
SHR1	10.903	3.401–34.954	0.000	14.639	4.075–52.589	0.000
SHR1≥0.98	2.792	1.647–4.731	0.000	2.842	1.595–5.065	0.000
SHR2	8.054	2.790–30.789	0.000	19.700	4.475–86.722	0.000
SHR3	1.864	0.729–4.763	0.194	2.111	0.781–5.705	0.141
ENI						
SHR1	0.133	0.028–0.621	0.010	0.086	0.014–0.510	0.007
SHR1≥0.98	0.413	0.212–0.804	0.009	0.332	0.155–0.710	0.004
SHR2	0.155	0.028–0.869	0.034	0.082	0.011–0.600	0.014
SHR3	1.014	0.354–2.901	0.979	0.931	0.288–3.007	0.905
Death						
SHR1	12.208	2.450–60.838	0.002	32.317	4.542–229.915	0.001
SHR1≥0.98	2.063	0.818–5.205	0.125	3.387	1.093–10.497	0.035
SHR2	11.790	2.333–49.488	0.003	88.767	7.087–1111.859	0.001
SHR3	1.765	0.329–9.460	0.507	2.992	0.372–24.049	0.303
Presence of hemorrhagic transformation						
SHR1	9.988	3.205–31.129	0.000	9.521	2.564–35.350	0.001
SHR1≥0.98	3.038	1.635–5.645	0.000	3.074	1.504–6.283	0.002
SHR2	10.342	3.885–60.013	0.000	16.622	3.310–83.464	0.001
SHR3	2.673	0.910–7.848	0.074	2.219	0.693–7.103	0.179
Presence of SICH						
SHR1	12.237	2.128–70.359	0.005	15.187	1.936–119.150	0.010
SHR1≥0.98	2.897	0.754–11.133	0.121	4.323	0.941–19.859	0.060
SHR2	18.703	2.118–212.356	0.010	28.418	1.966–410.681	0.014
SHR3	1.246	0.113–13.737	0.857	1.306	0.093–18.292	0.843

*Model 1 **was** adjusted for age, sex, and NIHSS*.

*Model 2 **was** adjusted for the variables included in Model 1 plus the following variables: hypertension, smoking, previous stroke/TIA, atrial fibrillation, diabetes, hyperlipidemia, systolic BP, diastolic BP, RBC, and PLT, which were identified as having P-values of <0.2 in Tables 1, 3*.

*SHR**, s**tress hyperglycemia ratio; NIHSS, National Institute of Health Stroke Scale; SICH, symptomatic intracerebral hemorrhage; TIA, transient ischemic attack; BP, blood pressure; RBC, red blood cell; PLT, platelet; OR, odds ratio; CI, confidence interval*.

## Discussion

Our study showed that the stress hyperglycemia ratio, as defined by SHR1 and SHR2, was associated with an elevated risk of worse outcomes in patients with ischemic stroke after intravenous thrombolysis. For the first time, we compared all definitions of SHRs from previous studies in our study (16, 18, 19, 24, 25). The present study found that SHR1 calculated by fasting glucose-to-HbA1c ratio has a better predictive performance for worse outcomes than other SHR definitions. Furthermore, multivariate logistic regression analysis showed that SHR1 and SHR2 were independently associated with ENI, death during the 3-month follow-up, and intracerebral hemorrhage but not SHR3.

HbA1c was considered as the average glycemia level over the past 8–12 weeks, representing the mean level of baseline blood glucose before stroke (26). Several studies have suggested that HbA1c and fasting glucose predicted a higher risk of worse clinical outcomes in patients with ischemic stroke undergoing intravenous thrombolysis (27, 28). In the present study, SHR was evaluated using different equations (16, 18, 19), which involved the extent of the acute elevation in plasma glucose based on the baseline glucose level prior to stroke. Our study showed that SHR1, which is defined as the ratio of fasting glucose to HbA1c, proved to have a better predictive ability for poor outcomes than HbA1c and fasting glucose alone in ROC analyses, as well as other SHR definitions.

Hyperglycemia is an independent predictor of unfavorable clinical outcomes in patients with ischemic stroke treated with intravenous thrombolysis (9, 12). However, the relationship between hyperglycemia and poor outcomes after ischemic stroke in patients receiving intravenous thrombolysis is controversial. A study carried out in Greece revealed that NIHSS at admission attenuates the risk of poor outcomes associated with hyperglycemia (29). In our study, the severity of stress hyperglycemia was associated with increasing stroke severity based on the NIHSS score. We adjusted for stroke severity at admission in the multivariate regression models, which did not weaken the risk of poor outcomes associated with SHR. Our study indicated that SHR was not only associated with the activation of the stress response but was also independently associated with an increased risk of poor functional outcomes. We speculate that SHR may synthetically reflect the degree of acute and chronic hyperglycemia, suggesting that the association between acute hyperglycemia and poor outcome risk may be a result of the long-term vascular destruction attributed to chronic hyperglycemia rather than acute hyperglycemia alone (27).

The results of our study were partly in line with the findings of previous studies (16, 17, and 24), confirming that SHR1 is a marker of increased risk of worse outcomes in patients with ischemic stroke receiving intravenous thrombolysis. We performed SHR2 as the stress hyperglycemia ratio and was predictive of worse outcomes, which was also presented in another study on mechanical thrombectomy for ischemic stroke (18). However, many studies carried out in both ICU and non-ICU patients indicate that SHR3 increased the likelihood of adverse outcomes (19, 30), which is contrary to the findings of our study. Tatiana et al. found that ENI could accurately predict vascular recanalization and prognosis after thrombolytic therapy (22). We found that SHR1 and SHR2 were inversely associated with ENI, indicating that higher SHR may be associated with lower vascular recanalization. To our knowledge, this is the first study to report a comparison of different definitions of SHRs associated with poor outcomes in patients with ischemic stroke after intravenous thrombolysis. Thus, our study contributes greatly to the available literature on stroke by consolidating the current concepts in public knowledge.

According to a previous study, SHR1 predicted hemorrhagic transformation in patients with non-thrombolysis ischemic stroke (25). However, few studies have investigated the relationship between SHRs and all-cause mortality. Our study showed that stress hyperglycemia, as measured by SHR1 and SHR2, was independently associated with **an increased risk of** death during the 3-month follow-up and SICH. Stress hyperglycemia represents an intense inflammatory response resulting from critical events such as acute stroke (31–33), which may exacerbate endothelial dysfunction and oxidative stress (34, 35). Hyperglycemia following ischemic stroke may increase free radical production and matrix metalloproteinase-9 activity, contributing to blood-brain barrier dysfunction (36).

This study had several limitations. First, the NIHSS score at admission, follow-up data, and SHR were not available in the 27 patients included in the study. However, no significant differences in baseline demography and disease characteristics were observed between the excluded and included participants. Second, only 29.3% of the patients were females, which may not be generalizable to other clinical centers. Third, this was a retrospective, single-center observational study with its inherent limitations, which indicates that multicenter clinical studies are necessary. Fourth, serum glucose was only collected at three time points in this study. Future prospective studies should focus on stress hyperglycemia fluctuations during follow-up.

## Conclusions

In conclusion, our study demonstrated that the stress hyperglycemia ratio, as measured by SHR1 and SHR2, was independently associated with worse clinical outcomes in patients with ischemic stroke after intravenous thrombolysis. Furthermore, SHR1 has a better predictive performance for outcomes than other SHR definitions. None of the randomized controlled clinical trials demonstrated that intensive glucose control enhances functional outcomes in patients with ischemic stroke and hyperglycemia. Then, we provide SHR instead of the absolute plasma glucose to become a potential treatment target for clinical trials.

## Data Availability Statement

The raw data supporting the conclusions of this article will be made available by the authors, without undue reservation.

## Ethics Statement

The studies involving human participants were reviewed and approved by the Ethics Committee of the First Affiliated Hospital of Wenzhou Medical University. The patients/participants provided their written informed consent to participate in this study.

## Author Contributions

C-LS and W-LZ conceived, designed the study, and wrote the manuscript. C-LS and HW organized the database. N-GX performed the statistical analysis. C-LS, N-GX, and HW reviewed and edited the manuscript. All authors read and approved the manuscript.

## Funding

This study was supported by the Wenzhou Science and Technology Bureau (No. Y20210913 and Y20180632) and Natural Science Foundation of Zhejiang Province (No. LQ21H090018).

## Conflict of Interest

The authors declare that the research was conducted in the absence of any commercial or financial relationships that could be construed as a potential conflict of interest.

## Publisher's Note

All claims expressed in this article are solely those of the authors and do not necessarily represent those of their affiliated organizations, or those of the publisher, the editors and the reviewers. Any product that may be evaluated in this article, or claim that may be made by its manufacturer, is not guaranteed or endorsed by the publisher.
