# Supplementary material for: Association of Stress Hyperglycemia Ratio With Acute Ischemic Stroke Outcomes Post-thrombolysis
Source: Front Neurol. 2022 Jan 13;12:785428. doi: 10.3389/fneur.2021.785428 (PMC8793935; doi:10.3389/fneur.2021.785428)
Supplement: Supplementary file 1 [file Table_1.pdf]

Table S1: Comparison of the baseline characteristics among **the** normal group, good outcome group, and poor outcome group

Characteristics	Normal group (n =73)	Good outcome (n =214)	Poor outcome (n =127)	P
Age (y), mean±SD	64.4±10.6	63.3±11.8	71.6±12.1	0.000
Gender, male, n (%)	47(64.4)	157 (73.4)	84 (66.1)	0.214
Baseline NIHSS score, median (IQR)	-	6 (3, 9)	11 (7, 15)	0.000
24 h NIHSS score, median (IQR)	-	3 (2, 6)	11 (7,16)	0.000
History of smoking, n (%)	32 (43.8)	98 (45.8)	41 (32.3)	0.044
Coronary artery disease, n (%)	0（0)	21 (9.8)	15 (11.8)	0.012
Hypertension, n (%)	56 (76.7)	134 (62.6)	95(74.8)	0.018
Diabetes, n (%)	26 (35.6)	49 (22.9)	28 (22.0)	0.064
Hyperlipidemia, n (%)	38 (52.1)	69 (32.2)	50 (39.4)	0.010
Previous stroke/TIA, n (%)	0（0)	23 (10.7)	18 (14.2)	0.005
Atrial fibrillation, n (%)	6 (8.2)	38 (17.8)	47 (37.0)	0.000
Systolic BP (mmHg), mean ± SD	151.0±22.0	153.7±22.4	158.1±24.5	0.078
Diastolic BP (mmHg), mean ± SD	82.0±13.1	86.2±14.9	85.9±15.7	0.101
Admission blood glucose (mmol/L), mean ± SD	-	7.7±3.1	8.3±3.2	0.098
Fasting plasma glucose (mmol/L), mean ± SD	5.5±1.9	5.8±2.1	6.9±3.0	0.000
2-hour postprandial blood glucose	8.5±4.0	8.3±3.5	8.5±3.2	0.872

HbA1c (%), mean ± SD	6.5±1.7	6.3±1.4	6.5±1.6	0.265
SHR1, mean ± SD	0.83±0.18	0.91±0.19	1.05±0.29	0.000
SHR2, mean ± SD	0.71±0.15	0.78±0.16	0.90±0.23	0.000
SHR3, mean ± SD	-	1.05±0.25	1.09±0.28	0.154
Onset to treatment time, min, mean ± SD	-	202.4±63.0	206.3±63.1	0.583
Cell count at admission				
WBC, x10^9/L, mean± SD	6.3±1.4	8.1±2.8	8.1±2.7	0.000
RBC, x10^12/L, mean± SD	4.4±0.5	4.6±0.5	4.5±0.6	0.267
PLT, x10^9/L, mean± SD	213.2±52.6	214.4±68.1	203.4±65.4	0.675

Abbreviations: NIHSS, National Institute of Health Stroke Scale; TIA, transient ischemic attack; BP, blood pressure; HbA1c, glycated hemoglobin; SHR, stress hyperglycemia ratio; WBC, white blood cell; RBC, red blood cell; PLT, platelet; SD, standard deviation; IQR, interquartile range.
